# Spectral Verification of the Mechanisms behind FBG-Based Ultrasonic Guided Wave Detection

**DOI:** 10.3390/s20226571

**Published:** 2020-11-17

**Authors:** Sidney Goossens, Francis Berghmans, Thomas Geernaert

**Affiliations:** Brussels Photonics (B-PHOT), Department of Applied Physics and Photonics, Vrije Universiteit Brussel and Flanders Make, Pleinlaan 2, B-1050 Brussels, Belgium; francis.berghmans@vub.be (F.B.); thomas.geernaert@vub.be (T.G.)

**Keywords:** fiber Bragg grating (FBG), ultrasonic guided wave (UGW), Lamb wave, spectral verification

## Abstract

Ultrasonic guided wave (UGW) detection with fiber Bragg grating (FBG)-based sensors has received increasing attention in the last decades due to the ability to perform non-destructive inspection (NDI) of large plate-like surfaces with a network of lightweight and multiplexed sensors. For accurate UGW measurements, several studies concluded that the ratio between the wavelength of the UGW and the length of the FBG should be above 7. However, shorter FBGs suffer from a lower FBG reflectivity and less steep slopes in the reflection spectrum. In this work we experimentally verified the effect of a passing UGW on the Bragg peak of FBG sensors of different lengths. By performing edge-filtering interrogation throughout the FBG’s reflection spectrum, we were able to reconstruct the FBG’s spectral response to a UGW in function of time. Our experimental findings are partially in line with those in the literature considering the UGW wavelength to FBG length ratio and the corresponding Bragg peak changes. We experimentally show for the first time that for shorter FBG sensors, the strain modulation is translated mostly into Bragg peak shifting, while for longer FBG sensors, Bragg peak deformation takes over as main mechanism. Despite the different mechanism for the latter, the UGW can still be detected by edge-filtering on the steepest slope, and with a much higher sensitivity.

## 1. Introduction

Ultrasonic guided wave (UGW) detection with optical fiber sensors has received interest in the last decennia due to the ability to investigate larger plate-like surfaces for damage with a lightweight, embeddable, EMI-immune and multiplexed network of sensors [1,2,3,4,5,6,7,8]. In a plate-like structure, actuated UGWs degenerate into Lamb waves with a typical wavelength of a few cm, depending on the actuation frequency [9]. Although fiber Bragg grating (FBG) sensors are often considered as point sensors, they have a physical length in the order of mm up to more than a cm. The ideal length of the grating versus the wavelength of the UGW has been investigated by several studies.

Coppola et al. [10] and Minardo et al. [11] identified three main working regions: when the wavelength of the UGW (λ_UGW_) is much smaller than the length of the grating (L), when λ_UGW_ is of the same order as L, and when the guided wave’s wavelength is much larger than the grating length. They showed by simulation that in the first region (λ_UGW_/L ≪ 1), a periodic positive and negative strain is acting along the length of the grating, yielding no net Bragg peak shift (Δλ_B_) and no change in the shape of the Bragg peaks. The second region (λ_UGW_/L ≈ 1) is a transition zone where partial Bragg wavelength shifts and peak chirp are generated by the UGW. Minardo et al. [11] additionally showed that in the transition region (λ_UGW_/L ≈ 1) the wavelength sensitivity of the Bragg peak (S_λ_) is proportional to the factor λ_UGW_/L. In the third region (λ_UGW_/L ≫ 1), the strain applied over the length of the FBG by the UGW is uniform, and thus, the complete Bragg peak shifts proportionally to the amplitude of the UGW: Δλ_B_ ~ A_UGW_ without deformation of the shape of the peak.

**Figure 1:**
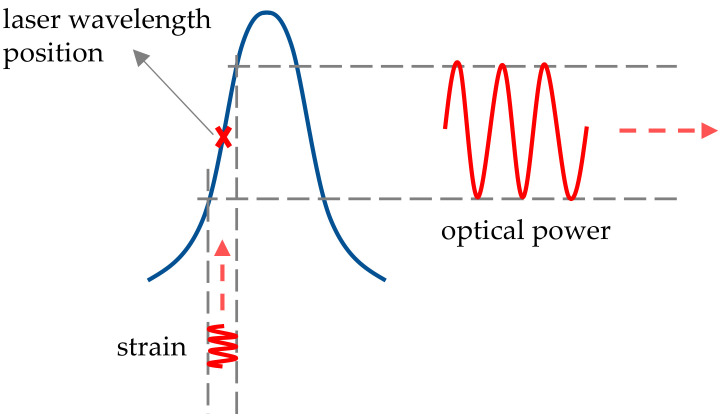
Edge-filtering fiber Bragg grating (FBG) interrogation: a narrow linewidth laser is set at the steepest slope of a Bragg peak. When a strain signal shifts (or deforms) the Bragg peak, the reflected optical power will change accordingly.

These regions were confirmed by Takeda et al. [4], Betz et al. [12], Culshaw et al. [1] and Thursby et al. [13]. It was generally stated that in order to yield a Bragg peak shift (Δλ_B_) modulation fully proportional to the strain generated by the passing UGW, a measurement should be performed in the region where λ_UGW_/L ≫ 1, as a uniform strain is then acting on the entire length of the FBG. In order to consider the strain sufficiently uniform, Takeda et al. [4] and Culshaw et al. [13] numerically determined that the respective conditions of λ_UGW_/L > 7 and λ_UGW_/L > 6 needed to be satisfied. These thresholds have been accepted as an important requirement for accurate UGW detection [2,5,14,15,16]. Since the wavelengths of UGWs around the resonance frequency of a plate-like structure are typically of the order of a few cm, this implies that the FBG sensor should be no longer than a few mm, depending on the exact application. Shorter FBG sensors, however, yield lower reflection levels compared to longer gratings manufactured by the same inscription technique [17]. Moreover, because most UGW interrogation of FBG sensors occurs by edge-filtering techniques, the sensitivity of the measurement depends of the slope of the linear part of the rising or falling edge of the Bragg peak. In edge-filtering, the wavelength of a narrow bandwidth laser is set at the location of the steepest slope of the reflection spectrum of an FBG as shown in Figure 1. When the Bragg peak is shifted (or deformed) due to a strain signal, the reflected optical power will change accordingly. Shorter FBG sensors, thus, inherently imply lower sensitivities for edge detection, which in its turn implies a lower signal-to-noise ratio, therefore requiring improved filtering and averaging to retrieve the UGW signal. Research has continued to investigate ways to increase the sensitivity of FBG sensors for UGW detection [16,18,19,20,21,22,23].

In this work, we attempted to experimentally validate the effect of different grating lengths on the acquired UGWs by performing an experimental spectral reconstruction of the Bragg peak during the passage of the UGW. This is the first analysis of its kind and gives experimental insights in the effect a UGW has on the FBG’s Bragg peak. From the reconstructed Bragg peak, the Bragg wavelength shift and peak broadening are obtained in function of time for different FBG lengths. From this information, the regions of λ_UGW_/L could be experimentally verified. Although we show that two different mechanisms contribute to the measurement signals acquired through edge-filtering for different λ_UGW_/L ratios, the mechanism itself does not seem that important for the validity of the measurement.

This manuscript is structured as follows: in Section 2, the experimental setup, including actuators and sensors, is explained, together with the methods used to actuate and acquire the UGWs. In Section 3, the resulting Bragg peak reconstruction during a UGW and the corresponding analysis are presented. In Section 4, the main observations from the experimental verification are discussed, followed by the conclusions. A link to the raw and processed data to reconstruct these results can be found in the Supplementary Materials section.

## 2. Materials and Methods

**Figure 2:**
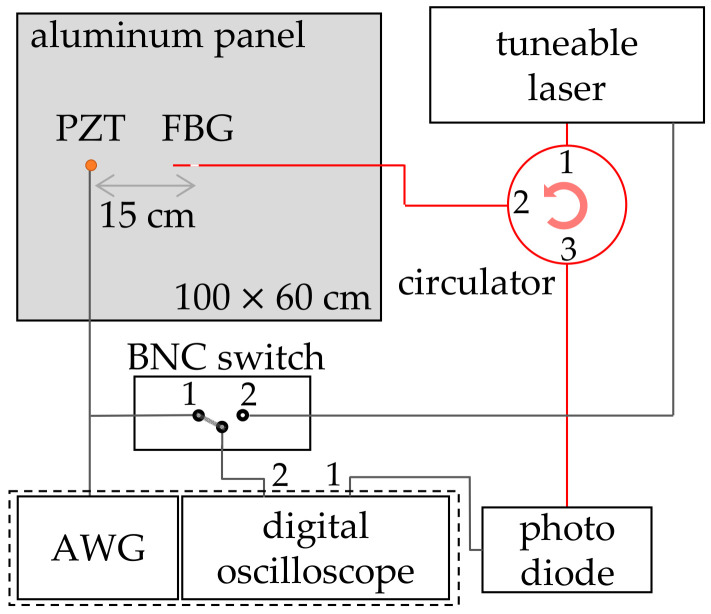
Experimental setup. Black lines represent electric BNC connections, red lines optical fiber connections.

We used an aluminum plate with dimensions of 1000 × 600 × 1 mm as our plate-like structure. These dimensions are large enough to avoid reflections of the UGWs from the sides during the acquisition of the first wave packet. The plate rested on four Styrofoam supports at every corner to approximate free-free boundary conditions by having a similar impedance as the air surrounding the rest of the plate. A schematic representation of the setup can be seen in Figure 2. The FBG under investigation was mounted in the center of the aluminum panel, and a PI Ceramics PRYY+0227 piezo transducer (PZT) [24] was mounted 15 cm from the FBG’s center. Both PZT and FBG were bonded with phenethyl salicylate soft glue, which allowed easy placement and removal by liquifying the substance with a heat gun. The FBG was prestrained by Kapton tape before the soft glue was solidified.

**Figure 3:**
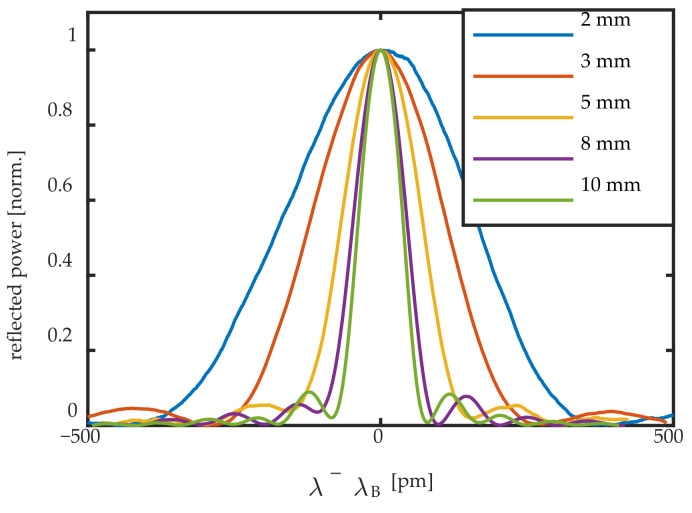
Normalized reflection spectrum of the five FBG sensors with increasing grating lengths.

Five FBG sensors with different length were surface mounted one by one for the measurements. The FBG sensors used in this work were standard uniform draw-tower-gratings (DTGs) in 125 μm diameter fiber with a GeO_2_-doped photosensitive core coated with Ormocer [25]. These FBG are of the type I, and can be considered weak FBGs [17]. Their properties are summarized in Table 1, and their (normalized) reflection spectrum can be seen in Figure 3. Note that increasing FBG lengths indeed yield higher reflection levels. In Figure 3, the effect of the FBG length on the slope of the reflection spectrum can be seen. Longer FBG sensors yield steeper slopes, and thus higher edge-filtering sensitivity.

The spectrum of the FBG sensor under investigation was acquired prior to every UGW measurement by the setup in Figure 2. While a wavelength range of 1 nm around the FBG was scanned by the Santec TSL-710 tunable laser source [26], the reflection was recorded by a Thorlabs PDA20CS InGaAs photodiode [27], which was sampled by the first channel of a TiePie HS5 digital oscilloscope [28]. Simultaneously, the trigger signal of the laser, which generated a 2 V peak every 100 pm, was acquired by the second channel of the oscilloscope. Together with the knowledge of the start wavelength, stop wavelength, laser scanning speed and recorded trigger signal, the reflection spectrum was reconstructed with a resolution of 0.4 pm. The Bragg wavelength was derived with a Gaussian fit.

The UGWs were generated by actuating the PZT with the TiePie HS5’s arbitrary waveguide generator (AWG) with a five-cycle Hamming-windowed sinusoid with a maximum amplitude of 12 V. The actuation signal was simultaneously recorded by the second channel of the digital oscilloscope and was used as a time trigger for the generated UGWs. At each spectral point, two UGW signals were generated: one at 50 kHz and one at 250 kHz, around the resonance frequencies of the antisymmetric and symmetric Lamb wave modes, respectively. The associated wavelengths of the UGWs in the aluminum plate were experimentally determined to be 18.6 mm for the 250 kHz and 31.9 mm for the 50 kHz signals.

For the FBG lengths used in this work, this resulted in λ_UGW_/L ratios between 2 and 16, as shown in Table 1. The buffer memory of the digital oscilloscope allowed for acquiring two five-cycle bursts separated 20 ms in time. The voltage output of the transimpedance amplifier of the photodiode was sampled at 10 MHz for 40 ms with a pretrigger of 10%. The two identical bursts were used to average two recorded signals, and the separation of 20 ms was required for the attenuation of all reflecting UGWs of the first burst. The UGW signals were recorded AC-coupled for higher amplitude resolution, but the DC offset of the spectrum at the corresponding wavelength was recorded prior to the UGW measurement.

**Figure 4:**
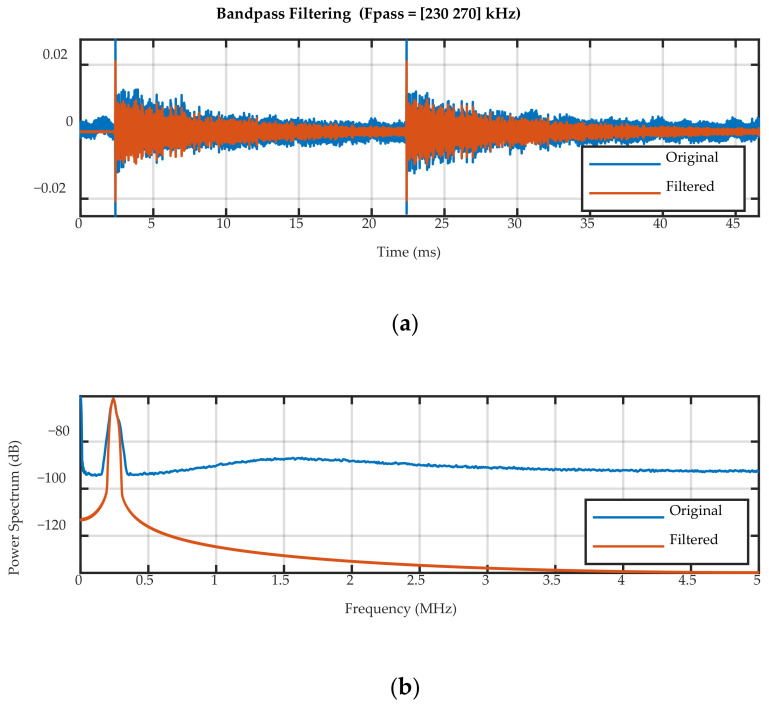
Bandpass filtering of a 250 kHz UGW acquired by the 10-mm FBG: (**a**) the unfiltered and filtered (unaveraged) 2 burst signal, and (**b**) the power spectral density of the unfiltered and filtered signals.

The recorded UGW signals were filtered with a bandpass filter to remove the high frequency laser noise and a 1 kHz oscillation on the laser power. The pass frequencies for the 50 kHz signal were 30 and 70 kHz, and for the 250 kHz signal they were 230 and 270 kHz. An example of the filter applied to a 250 kHz UGW acquired by the 10-mm FBG is shown in Figure 4. After filtering, the UGW signals were averaged over the two bursts.

**Figure 5:**
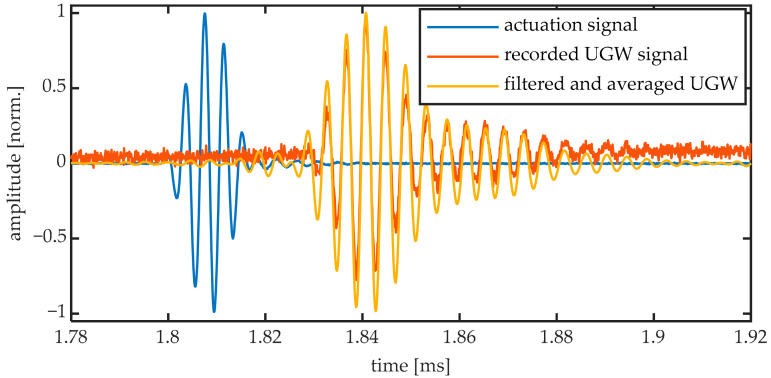
Actuation signal as measured by the oscilloscope, UGW signal as measured by the photodiode and filtered and average UGW signal of a 250 kHz UGW acquired by the 10-mm FBG.

A Hilbert transform was performed on the directly measured actuation signal to obtain the Hamming window shape from the five-cycle burst, which was then fit by half a period of a square sinusoid. From this fit, the start of the burst was calculated and used for synchronizing and averaging both bursts. An actuation signal and UGW signal acquired by the digital oscilloscope can be seen in Figure 5, together with the resulting filtered and averaged signal that was used for further processing. From the maxima of the Hamming fit, we determined the time delay between the actuation and the arrival of the first wave packet to be 95.3 ± 0.3 μs for the 50 kHz signals and 32.7 ± 0.1 μs for the 250 kHz signals. This corresponds to group velocities of 1595 m/s and 4654 m/s for the main wave packets obtained at 50 kHz and 250 kHz, respectively. These velocities correspond to the group velocities of the fundamental antisymmetric and symmetric mode, respectively, for aluminum.

The acquired signal in Figure 5 shows more than five cycles of a sinusoid. This can be explained by the generation of additional modes that travel at different velocities. These additional modes are, however, typically ignored in further analysis by focusing only on the predominant wave packet of the resonant mode [29]. We will, therefore, similarly focus our analysis only on the (amplitude of the) predominant wave packet.

## 3. Results

**Figure 6:**
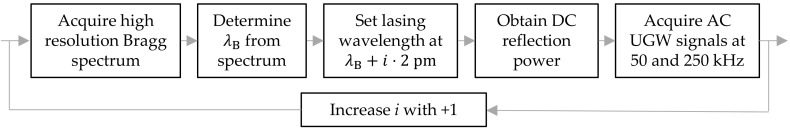
Block diagram showing the steps of the spectral reconstruction of the Bragg peak during UGW passage.

**Figure 7:**
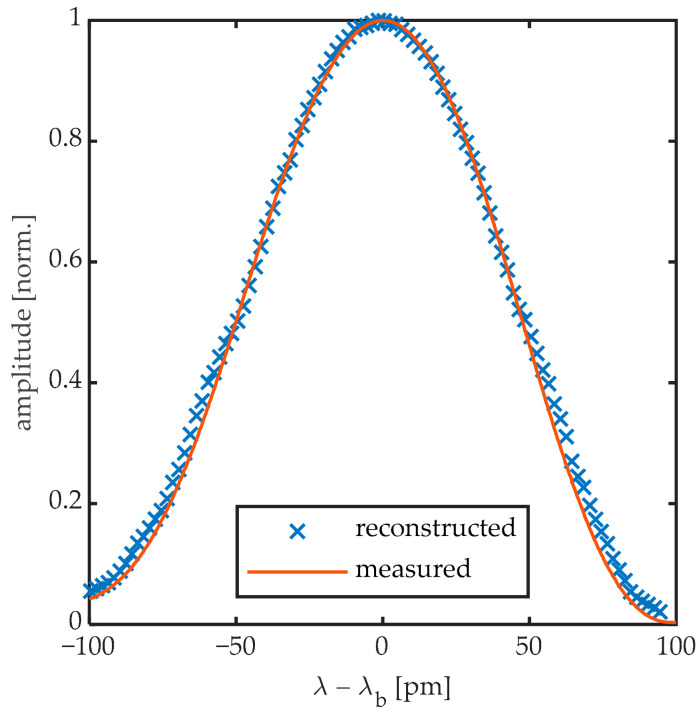
Initially measured reflection spectrum compared to the stepwise reconstructed spectrum at t = 0 obtained from the UGW offset signals.

In order to reconstruct the dynamics of the Bragg peak during the passage of a UGW, the UGW signal was obtained by edge-filtering not only at the steepest slope locations of the Bragg peak, as is typically done, shown in Figure 1, but at a multitude of wavelengths along the Bragg peak’s reflection spectrum. To do so, we followed the steps in the block diagram shown in Figure 6. Prior to every UGW measurement, the spectrum of the FBG was acquired and the Bragg wavelength of the spectrum was calculated. Then, the lasing wavelength was set at a specific location on the spectrum relative to $\lambda_{B}$. Once the laser was positioned at that wavelength, the static DC component of the reflected optical power was recorded. Figure 7 shows the high-resolution spectrum of the 8 mm DTG obtained prior to the first UGW, together with the individually determined DC values obtained prior to every UGW measurement. By defining the lasing wavelength for each UGW measurement relative to $\lambda_{B}$, we considered possible temperature effects that could shift the Bragg peak over time. After the DC component is recorded, the laser remains at the same position for acquiring the AC signals of the UGWs actuated at 50 and sequentially at 250 kHz by the setup shown in Figure 2. This whole process is then repeated for the next laser wavelength as illustrated in Figure 6. The range of the parameter i depends on the bandwidth of the FBG under investigation. For the example of the 8-mm FBG in Figure 7, i ranged from −50 to +50, spanning a bandwidth of 200 pm around $\lambda_{B}$ with measurements every 2 pm.

**Figure 8:**
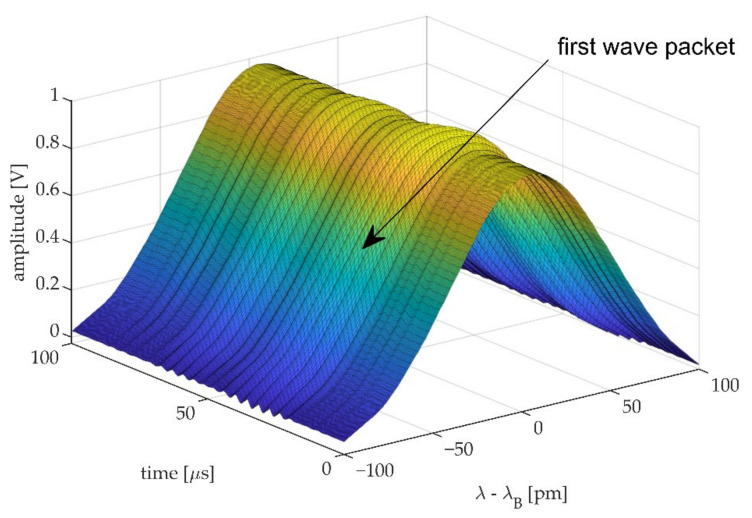
Full spectral reconstruction of 250 kHz UGW passing through an 8-mm FBGs. AC amplitude of the UGWs is artificially increased by a factor 10 for visualization.

By adding the DC component obtained prior to the UGW measurement, back to the AC coupled UGW measurement and with the knowledge of the spectral position relative to the Bragg wavelength, the dynamics of the Bragg peak can be reconstructed as shown in Figure 8. In this figure the amplitude of the UGWs is artificially increased by a factor 10 for visualization. Figure 8 shows a 250 kHz UGW obtained by the 8-mm FBG in the first 100 μs of acquisition. The first wave packet can be identified as the Bragg peak deforms in function of time.

**Figure 9:**
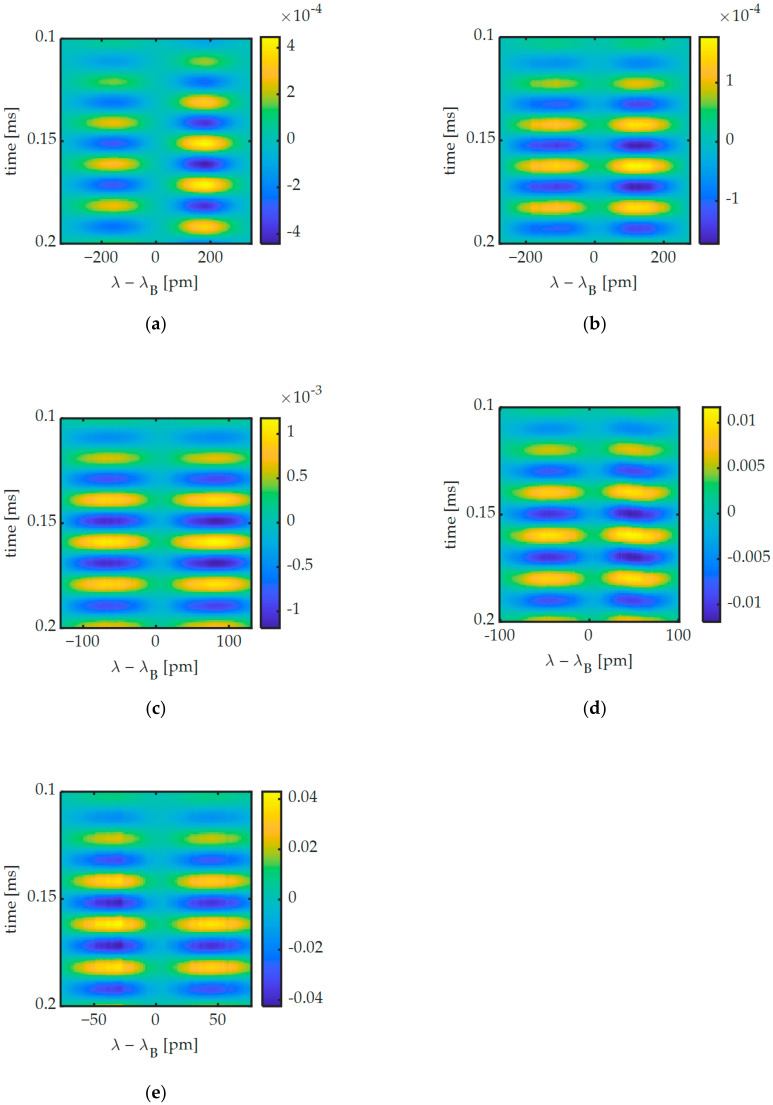
First wave packet of AC coupled 250 kHz UGW signals measured along the spectrum of (**a**) the 2-mm, (**b**) the 3-mm, (**c**) the 5-mm, (**d**) the 8-mm and (**e**) the 10-mm FBG.

Figure 9 shows the AC coupled UGW signals for all FBG lengths. In Figure 9a, it can be observed that during the first wave packet the signals on either slope of the Bragg peak are out of phase, implying that mostly Bragg peak shifting is occurring for the 2-mm FBG. This contrasts with the other FBGs shown in Figure 9b–e, where the signals are more in phase, implying Bragg peak broadening is occurring. This is a first indication that different mechanisms occur in FBGs of different length, which we will investigate in greater detail below.

**Figure 10:**
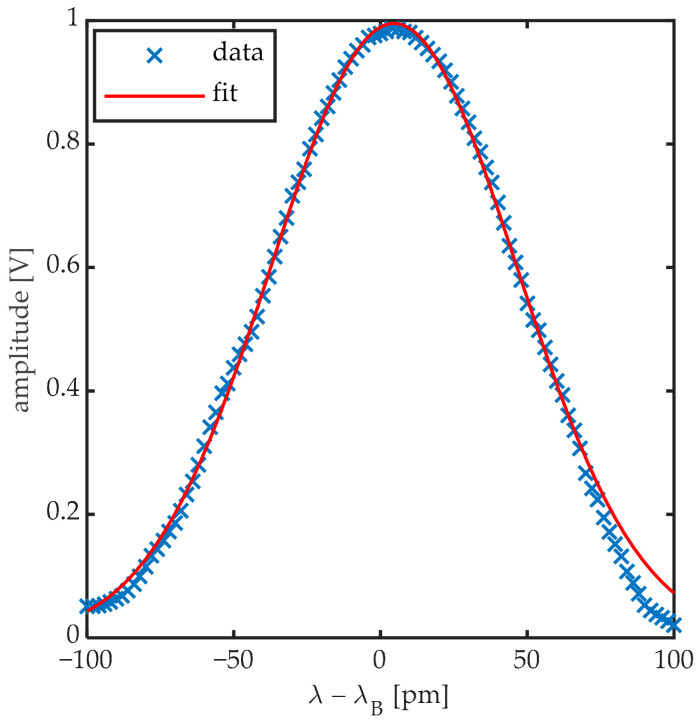
Locations on the spectrum of the 8-mm FBG where the laser was locked for performing UGW measurements at t = 0, together with the Gaussian fit used to extract the Bragg peak’s characteristics.

In order to obtain the Bragg wavelength change (Δλ_B_) and Bragg peak broadening due to the change in full width at half maximum (ΔFWHM), we performed a Gaussian fit of the reconstructed spectrum at every time step (0.1 μs). This is shown in Figure 10 for the first reconstructed spectrum of the 8-mm FBG of Figure 8. The Gaussian fit was performed with the peak’s amplitude values as weights, to obtain a higher fit correlation of the top half of the Bragg peak. From the Gaussian fit parameters, the Bragg wavelength and FWHM can be straightforwardly calculated.

**Figure 11:**
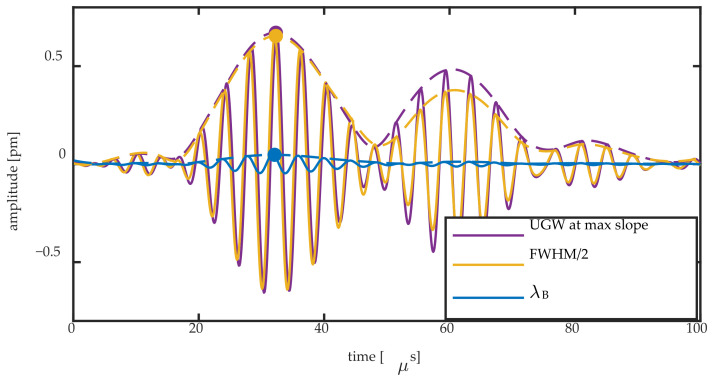
Bragg wavelength shift and FWHM/2 change of the 8-mm FBG during the passage of a 250 kHz UGW obtained by the Gaussian fit compared to the UGW measured at the steepest slope. The Hilbert transform of all signals is shown by a dashed line, and the maximum amplitude of the latter is shown by a full dot in the same color.

**Figure 12:**
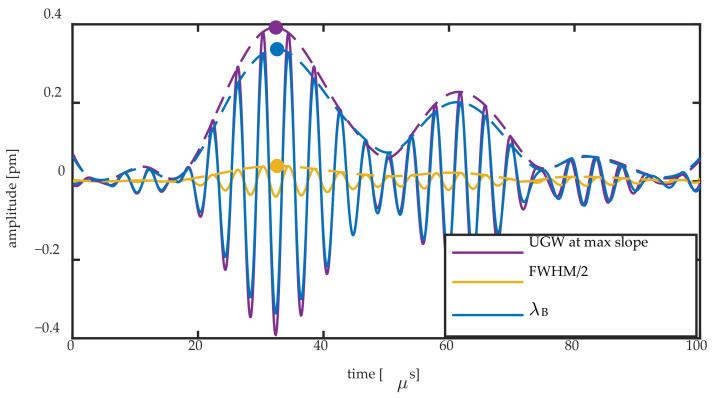
Bragg wavelength shift and FWHM/2 change of the 2-mm FBG during the passage of a 250 kHz UGW obtained by the Gaussian fit compared to the UGW measured at the steepest slope. The Hilbert transform of all signals is shown by a dashed line, and the maximum amplitude of the latter is shown by a full dot in the same color.

Figure 11 shows the Bragg wavelength change and FWHM/2 change obtained by the Gaussian fit for the 8-mm FBG during passage of a 250 kHz UGW, together with their Hilbert transform. The two signals are compared to the UGW obtained on the steepest slope of the Bragg peak. For the latter signal, the acquired voltage signal was divided by the slope of the Bragg peak at that location to obtain a signal in pm. The slope was obtained by performing a linear fit in an 8 pm region centering the spectral position of the measurement. Figure 12 shows the same signals for the 2-mm FBG.

## 4. Discussion

Figure 11 and Figure 12 show that there are two mechanisms contributing to the UGW that is acquired on the steepest slope of a Bragg peak: the peak shift and Bragg peak broadening. The former can be quantified directly by $\Delta \lambda_{B}$, and the latter can be partially quantified by ΔFWHM/2. Any non-symmetric peak deformation is not captured by this method. For an FBG of a longer length, the more pronounced mechanism is the peak broadening, as shown by the amplitude of the yellow signal that approaches that of the purple signal in Figure 11. However, for a shorter FBG length, the predominant mechanism is a shift of the entire Bragg peak, as shown by the amplitude of the blue signal approaching that of the purple signal in Figure 12. In order to relate the amplitudes of the signal to each other, we obtained the Hilbert transform of each signal as shown in the examples of Figure 11 and Figure 12. We used the maximum of each Hilbert transform at the first wave packet as quantification for the comparison of each signal’s amplitude, as denoted by the full dots in Figure 11 and Figure 12. This quantification allowed us to obtain the ratio of the amplitudes of Δλ_B_ and ΔFWHM/2 to the UGW signal acquired at the steepest slope.

**Figure 13:**
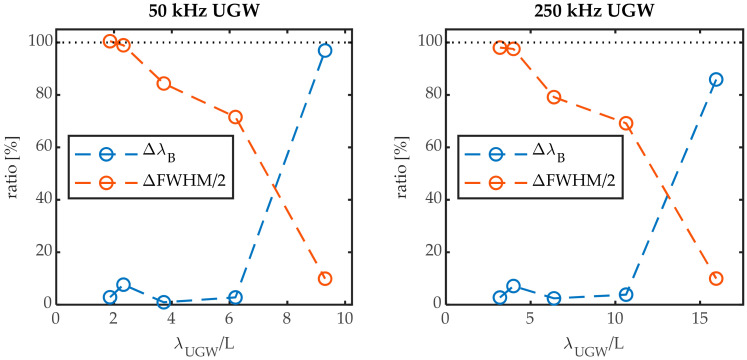
Ratio of the maximum amplitude of the Hilbert transform of the Bragg peak shift and Bragg peak broadening to that of the UGW measured at the steepest slope.

These ratios for the 50 kHz and the 250 kHz signals are shown in Figure 13 in function of the factor λ_UGW_/L. From Figure 13 we can observe that there are indeed two main mechanisms contributing to the UGW that was measured at the steepest slope of the Bragg peak: in the region where λ_UGW_/L approaches 1, Bragg peak broadening is responsible for ~100% of the movement at the steepest slope. This region is followed by a transition region as λ_UGW_/L increases: the contribution of (symmetric) peak broadening is no longer 100% and decreases with increasing λ_UGW_/L. The contribution of the peak shift Δλ_B_, however, remains low, implying antisymmetric peak deformation is occurring. At a certain λ_UGW_/L, the main contributing mechanism is no longer peak deformation, as the peak shift Δλ_B_ becomes the dominant mechanism. For the 50 kHz signal, this is around λ_UGW_/L ≈ 8, and for the 250 kHz signal this is around λ_UGW_/L ≈ 13. We, thus, notice a difference between the two frequencies. This can be attributed to the antisymmetric mode being the wave packet under investigation at 50 kHz, and the symmetric mode being the wave packet under investigation at 250 kHz. Either mode has different strain shapes at the surface of the plate, which interact differently with the FBG sensor.

For a factor λ_UGW_/L above these particular values of 8 and 13, the acquired signal at the steepest slope will thus be attributed to a predominant wavelength shift Δλ_B_. For a high enough λ_UGW_/L ≫ 1 the UGW amplitude is completely transcoded into a Bragg peak shift, and no Bragg peak deformation is present, implying that the UGW is applying only uniform strain over the length of the FBG sensor.

The transition region is centered around the ratios λ_UGW_/L = 6 or 7 as reported in literature; however, for a complete uniform strain over the FBG, the factor λ_UGW_/L from these experimental results for inducing only a Bragg peak shift is at λ_UGW_/L > 13 for the symmetric mode and λ_UGW_/L > 8 for the antisymmetric UGW mode, which is slightly larger than what was reported in literature [1,4]. For the FBG sensors used in this work, the only FBG sensor satisfying that condition was the FBG of L = 2 mm. As was shown in Table 1 and Figure 3, this sensor had a reflectivity about 10 times lower than that of the 10-mm FBG, with much less steep slopes, implying that the sensitivity for edge-filtering measurements is also much less.

**Figure 14:**
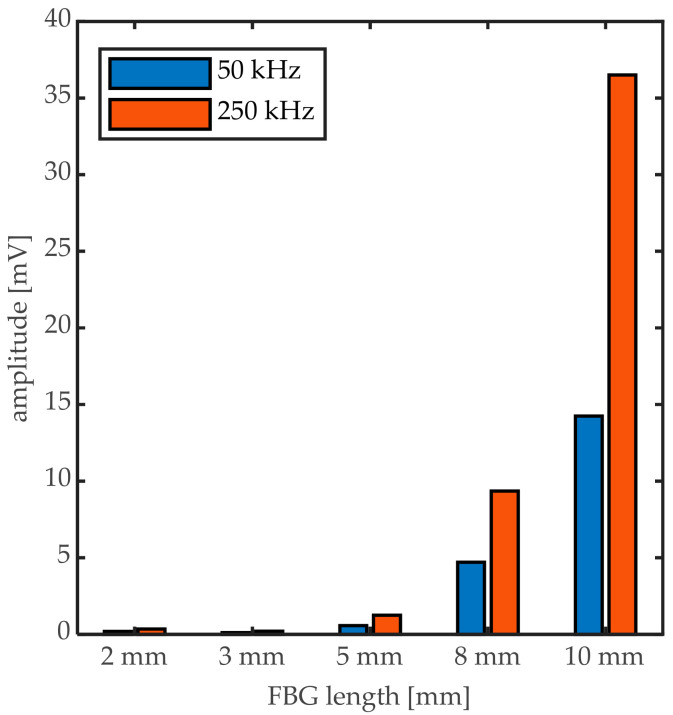
Absolute maximum amplitude of the UGWs measured by different FBG length.

This is confirmed by the absolute signal amplitudes measured by the different FBGs that are shown in Figure 14. The difference in amplitude between the 2-mm FBG and the 10-mm FBG is up to a factor 100 for the 250 kHz signal. This implies that using the shortest available FBG length could implicitly decrease the sensitivity of the measurement drastically. It is, however, often argued in the literature that only a UGW signal corresponding completely to a Bragg peak shift can be interpreted correctly as strain signal, and the requirement of λ_UGW_/L ≥ 1 must, thus, be fulfilled in order to obtain the full information of the UGW.

**Figure 15:**
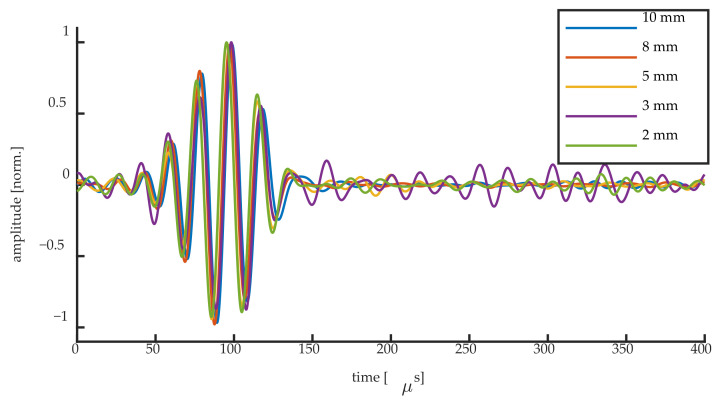
Normalized UGW signals obtained for all FBG lengths for the 50 kHz signals.

**Figure 16:**
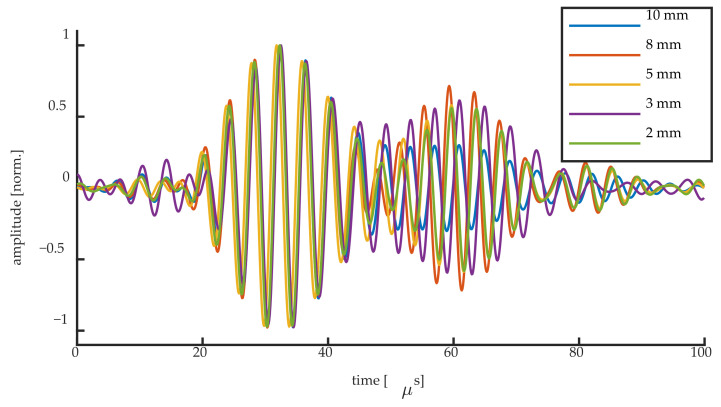
Normalized UGW signals obtained for all FBG lengths for the 250 kHz signals.

Figure 15 and Figure 16 show the normalized UGW signals acquired at the steepest slope location for the 50 kHz UGWs and the 250 kHz UGWs, respectively. Although we have shown that different mechanisms are responsible for the signals acquired at the steepest slope of the Bragg peak, the normalized signals of the first wave packet are identical for all grating lengths. This implies that even when the condition λ_UGW_/L ≥ 1 is not fulfilled, the UGW signals remain measurable at the steepest slope, even if they are generated by Bragg peak deformation instead of shifting. Opting for higher grating sensitivity might, thus, be preferred over opting for short FBG length. This applies in particular to weak FBGs, where longer gratings typically show a higher reflectivity and, thus, a higher sensitivity when considering edge detection [17], resulting in a higher SNR, thus requiring less signal filtering and averaging. In some studies the averaging of 100 up to 10,000 UGW signals is reported for noise reduction [8,12,30], which could significantly be decreased in the case of weak FBGs by using the higher sensitivity of a longer FBG sensor. Moreover, many applications of UGWs, such as damage detection, make use of the difference between UGW signals, where the mechanism generating the UGW signals is of no importance.

## 5. Conclusions

In this work we demonstrated for the first time a full spectral reconstruction of the Bragg peaks of different grating lengths during the passage of a UGW. We experimentally confirmed the effect of the ratio of the UGW’s wavelength (λ_UGW_) to the grating length (L) on the Bragg peak, as reported by simulations in the literature. For λ_UGW_/L ≥ 1, the strain acting on the FBG can be considered uniform and the amplitude of the UGW is transcoded completely in a Bragg peak shift, Δλ_B_. When λ_UGW_/L approaches unity, the strain over the FBG is no longer uniform, and peak broadening starts to occur (ΔFWHM). The condition for pure Δλ_B_ was found to be between λ_UGW_/L ≥ 13 and λ_UGW_/L ≥ 8 for the symmetric Lamb wave mode and anti-symmetric mode, respectively, which is slightly larger than what was previously reported in literature.

We showed however that, even when Bragg peak broadening becomes the dominant mechanism as λ_UGW_/L approaches unity, the shape of the first wave packet of the UGW signal acquired on the steepest slope of the Bragg peak remains identical to that of smaller FBG lengths. Moreover, increased FBG length implicitly implies higher Bragg peak reflectivity and slopes, which in its turn implies increased sensitivity when performing edge-filtering interrogation. Higher sensitivity implies that less filtering and averaging is required, increasing the quality of the measurement.

It is, therefore, not strictly required to fulfill the requirement of λ_UGW_/L ≫ 1, as is often assumed. Although Bragg peak broadening will be the dominant mechanism at increased FBG length, the UGW signal can still be acquired through edge-filtering. Moreover, the signal amplitude will be significantly larger for a longer FBG sensor and might, therefore, even be preferred.

## Figures and Tables

**Table 1:** Overview of FBG sensors and their properties. FWHM: full width at half maximum.

	FBG #1	FBG #2	FBG #3	FBG #4	FBG #5
L [mm]	2	3	5	8	10
FWHM [pm]	355	247	147	98	81
λ_B_ [nm]	1550	1555	1540	1570	1565
R [%]	3.6	5.5	15.3	33.4	36.9
λ_UGW_/L (50 kHz)	9.3	6.2	3.7	2.3	1.9
λ_UGW_/L (250 kHz)	15.9	10.6	6.4	4.0	3.2

## Supplementary Materials

The filtered and averaged UGW data and resulting Gaussian fit data are available at http://dx.doi.org/10.17632/wz59sf6ck8.1.
